# Running Economy from a Muscle Energetics Perspective

**DOI:** 10.3389/fphys.2017.00433

**Published:** 2017-06-22

**Authors:** Jared R. Fletcher, Brian R. MacIntosh

**Affiliations:** Human Performance Laboratory, Faculty of Kinesiology, University of CalgaryCalgary, AB, Canada

**Keywords:** energy cost, muscle contraction, oxygen consumption, respiratory exchanges, training-induced changes

## Abstract

The economy of running has traditionally been quantified from the mass-specific oxygen uptake; however, because fuel substrate usage varies with exercise intensity, it is more accurate to express running economy in units of metabolic energy. Fundamentally, the understanding of the major factors that influence the energy cost of running (E_run_) can be obtained with this approach. E_run_ is determined by the energy needed for skeletal muscle contraction. Here, we approach the study of E_run_ from that perspective. The amount of energy needed for skeletal muscle contraction is dependent on the force, duration, shortening, shortening velocity, and length of the muscle. These factors therefore dictate the energy cost of running. It is understood that some determinants of the energy cost of running are not trainable: environmental factors, surface characteristics, and certain anthropometric features. Other factors affecting E_run_ are altered by training: other anthropometric features, muscle and tendon properties, and running mechanics. Here, the key features that dictate the energy cost during distance running are reviewed in the context of skeletal muscle energetics.

## Introduction

### Importance of E_run_ to distance running performance

Endurance running performance is determined by a combination of physiological, anthropometric, and biomechanical factors. These factors include a high maximal oxygen uptake ($\dot{V}O_{2max}$), the ability to minimize disturbance to homeostasis while sustaining a higher fraction of $\dot{V}O_{2max}$ and a low energy cost to run (E_run_) at that high fraction of $\dot{V}O_{2max}.$ With few exceptions, world-class male marathon running performances are achieved by runners who possess $\dot{V}O_{2max}$ -values above 75 ml·kg^−1^·min^−1^ and the fraction of $\dot{V}O_{2max}$ that can be sustained for the marathon distance is at least 80% of $\dot{V}O_{2max}$ (Foster and Lucia, 2007). Using the American College of Sports Medicine's metabolic equations for the energy cost of running over level ground, a mean $\dot{V}O_{2}$ of 71.9 ml·kg^−1^·min^−1^ is required to achieve the current marathon world-best time of 2:02:57. Assuming this runner has a body mass of 56 kg and their respiratory exchange ratio is 0.95, this oxygen uptake would equate to an E_run_ of 4.39 J·kg^−1^·m^−1^. E_run_ values this low are frequently reported (Foster and Lucia, 2007; Fletcher et al., 2009; Shaw et al., 2013), but assuming the marathon distance could be sustained at 85% $\dot{V}O_{2max}$, this runner would require a $\dot{V}O_{2max}\;$ near 85 ml·kg^−1^·min^−1^. A marathoner, with an excellent E_run_ of 3.77 J·kg^−1^·m^−1^ (Fletcher et al., 2009) would only require a $\dot{V}O_{2max}$ of 77.5 ml·kg^−1^·min^−1^, so it is likely the runner who is going to break the sub-2 h marathon will be one with extraordinary E_run_. But how is an extraordinary E_run_ achieved?

**Figure 1:**
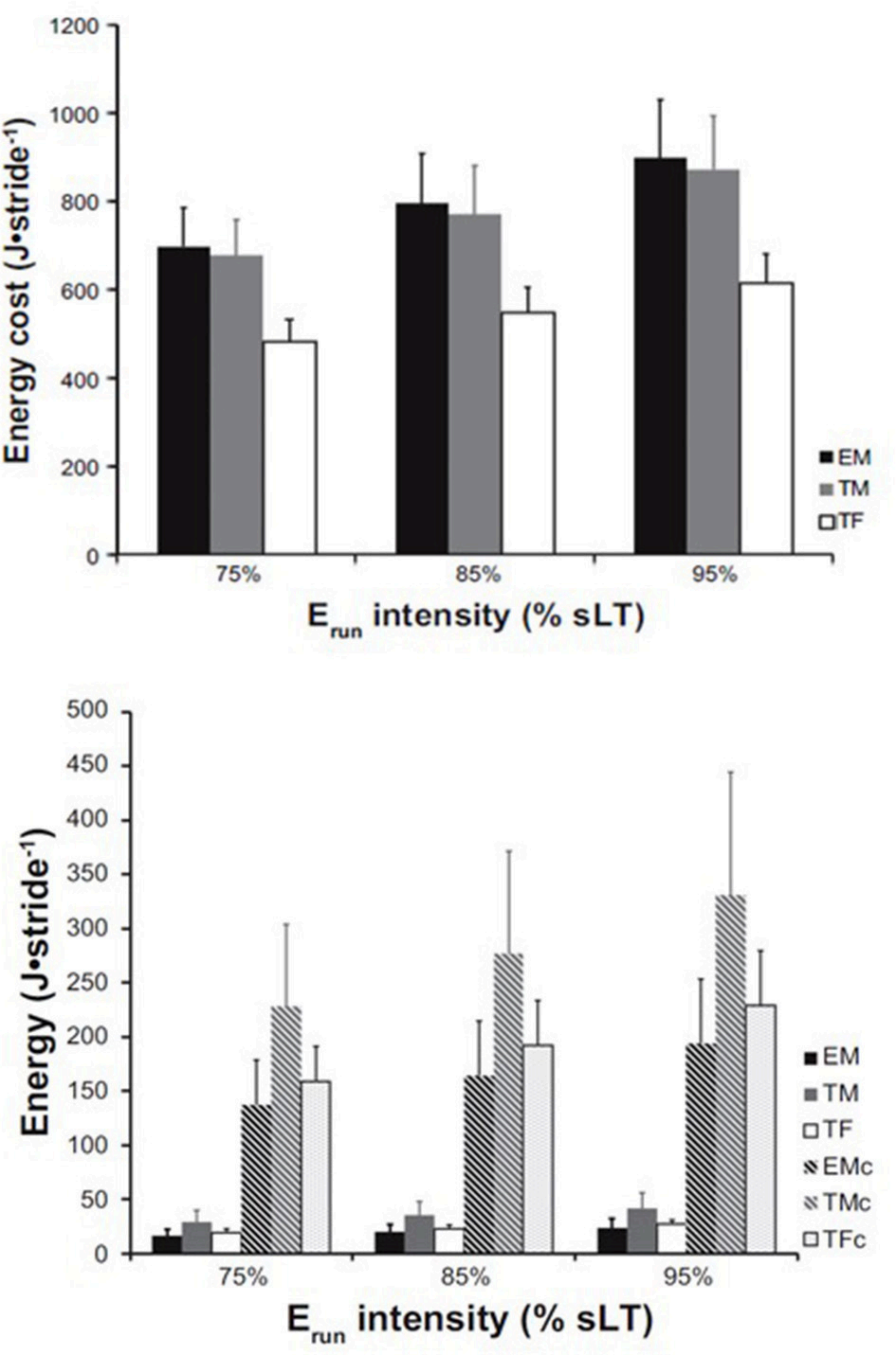
E_run_ and muscle energy cost in male and female runners. Whole-body energy cost per stride **(top)** estimated across three relative running speeds expressed as % of speed at lactate threshold in three groups: Elite males (EM), trained males (TM), and trained females (TF). **Bottom**: AT energy release (solid bars) relative to the estimated muscle energy cost (hatched bars) for each group and speed, respectively. EMc, TMc, and TFc represent the energy cost for elite males, trained males, and trained females, respectively. From Fletcher and MacIntosh (2015).

It is known that E_run_ is likely influenced by a number of physiological and biomechanical factors and several excellent reviews have been written on the topic in the last 25 years (Morgan et al., 1989; Morgan and Craib, 1992; Saunders et al., 2004; McCann and Higginson, 2008; Lacour and Bourdin, 2015). None of these reviews has approached E_run_ from a muscle energetics standpoint. Recently, we have estimated that the active skeletal muscle energy cost represents the vast majority of the total metabolic cost of running (Fletcher and MacIntosh, 2015). Specifically, we have estimated that the energy cost of triceps surae muscles contraction during the running stride of highly-trained runners represents nearly 25% of the total metabolic cost of running. This proportion increases to nearly 40% in lesser-trained male and female runners (Figure 1). The energy cost of other active muscles, of course, also contribute to the total metabolic cost of running. Consequently, probing the specific factors that dictate the muscle energy cost during running, which include running speed, body mass, and muscle-tendon mechanical and morphological properties (tendon stiffness, fascicle length) should provide unique insight into the underlying factors that determine E_run_ and may reveal the mechanisms behind changes in E_run_ with training, disuse or disease.

### Quantifying the energetics of running

ATP is resynthesized from ADP and Pi using the energy released during oxidative phosphorylation. O_2_ is consumed when it accepts electrons at the end of the electron transport chain to form ATP via ATP synthase. Thus, $\dot{V}O_{2}$ reflects the quantity of ATP used when aerobic metabolism can provide all of the energy at a given running speed. This is only true: (1) when sufficient time is given to achieve a physiological steady-state and (2) when the speed is less than that which results in accumulation of blood lactate. This latter point is important because at speeds greater than the anaerobic threshold, steady-state conditions are unlikely as a result of the $\dot{V}O_{2}$ slow component and non-aerobic metabolism contributes to the energy cost. Understanding skeletal muscle energetics will ultimately lead to a better understanding of E_run_.

## Skeletal muscle energetics

Without muscle contraction, running would be impossible. Here, we review the general factors that influence the energy cost of running, and try to put them into the context of understanding the role that muscle contraction and muscle energetics plays in contributing to variability in the E_run_. Muscle energy cost *in vivo* arises from cross-bridge turnover as well as the energy cost of ion pumping, primarily from the Na^+^-K^+^ ATPase and the sarco-endoplasmic reticulum Ca^2+^ ATPase (SERCA) pumps (Barclay, 2015). The energy cost associated with Ca^2+^ re-uptake represents the majority of the energy cost associated with ion pumping (Homsher and Kean, 1978).

Combining the energy cost of SERCA and Na^+^-K^+^ ATPase pumps accounts for 30–40% of the energy used during an isometric contraction, where the energy associated with cross-bridge cycling as a result of shortening is not considered (Homsher et al., 1972; Barclay et al., 1993; Barclay, 1996). When shortening occurs, the proportion of the energy cost attributed to non-cross-bridge ATPases becomes less because shortening considerably increases the cross-bridge turnover (Smith et al., 2005). Considering this energy cost of shortening, a fixed-end muscle contraction is more costly in the initial part of the contraction when force is rising because the fascicles shorten against the series elastic components of the muscle. During locomotion, most muscle contractions are of short duration (Novacheck, 1998), so this is a relevant aspect of the energy cost of running.

### Turnover of cross-bridges: isometric contractions

During an isometric contraction, energy cost is elevated compared to the resting state. Since, by definition no external work is performed during an isometric contraction, the energy cost must arise primarily from time-dependent cross-bridge cycling. Barclay et al. (2010a) have previously estimated this rate to be 1.5 ATP split·s^−1^ per cross-bridge in frog sartorius muscle at 0°C. In human quadriceps muscle at physiological temperature, assuming the ATP turnover rate is 1 mmol·kg^−1^ wet wt·s^−1^ (Katz et al., 1986), each cross-bridge splits 5.6 ATP ·s^−1^. This assumes that the ATP splitting cycle requires 180 ms. A cross-bridge duty cycle of 0.33 (Barclay et al., 2010a) would require the cross-bridge be attached for 60 ms; the remaining 120 ms presumably being required for the cross-bridge to return to a state from which it can attach again. This duty cycle would be fiber-type specific; a smaller duty cycle is associated with fast-twitch fibers. Thus, the energy required during an isometric contraction is dependent solely on the required force (which dictates the number of cross-bridges in parallel required to generate that force) and the contraction duration. However, recognizing that as force develops in a fixed-end contraction, the tendon is stretched thereby requiring shortening of the muscle fascicles, additional cross-bridge cycles will occur. Muscle length must also be considered. Each sarcomere in series adds to the energy needed for the muscle contraction because the same force must be generated in each half-sarcomere.

### Shortening contractions

Shortening at a velocity that requires a faster turnover than the isometric cross-bridge turnover rate can increase the rate of turnover of individual cross-bridges. This velocity is dependent on myosin isoform; fast-twitch myosin isoforms will reach this velocity at a slower shortening velocity because their isometric cross-bridge cycle is faster. This velocity beyond which the shortening energy cost exceeds the isometric cost is the equivalent of a cross-bridge sweep per isometric cross-bridge cycle time for each half sarcomere of fiber length. At the optimal velocity, that for which efficiency is maximal, the energy cost of a shortening contraction is 2–3-fold greater than that expected during an isometric contraction. This increase in energy demand is referred to as shortening-induced increase in ATP turnover (Woledge et al., 1988). The amount of ATP split, and therefore energy use by the muscle is increased in proportion to the amount of shortening within each half sarcomere and is dependent on the working stroke (or cross-bridge sweep) of each cross-bridge. Because the force per cross-bridge decreases with increasing velocity (de Tombe and Ter Keurs, 1990), increased activation is needed (more cross-bridges in parallel) to maintain the same force during shortening. Therefore, the shortening energy cost is also proportional to shortening velocity (Hill, 1938; Homsher et al., 1972).

### Energy cost of muscle contraction

Running can be considered a series of voluntary muscle contractions; the force of contraction being dictated by the running speed and the controlled motion of the lower leg. The required level of voluntary muscle activation is primarily determined by the force-length-velocity relationships of the muscle and the need for force or movement through a specific angular displacement. The level of muscle activation, a combination of motor unit recruitment and rate coding or increased frequency of activation of already active motor units (Fuglevand et al., 1993), dictates the energy cost since the rate of energy use depends on the number of fibers activated, the cross-bridge turnover rate, and the number of cross-bridge cycles required. The muscle volume-specific rate of energy use is greater in fast-twitch muscles during isometric contractions and slow shortening because faster muscles have higher rates of time-dependent cross-bridge cycling (Rall, 1985; Katz et al., 1986). However, once the velocity of shortening achieves a rate of ATP splitting that exceeds the isometric cross-bridge turnover rate for slow-twitch myosin, the fiber-type difference disappears because cross-bridges disengage as a result of fast shortening, not because of fiber-type dependent cross-bridge turnover rate.

The energetic cost of generating force is also dependent on the average length of the activated muscle fiber. For muscles having similar fiber type compositions and operating under similar levels of activation and shortening velocities (relative to length), muscles with shorter fascicles (fewer sarcomeres in series) can be expected to consume proportionally less ATP per unit force generated compared to muscles with longer fascicles (Roberts et al., 1998b). The volume of active muscle recruited to generate the required force is the product of fascicle length and active cross-sectional area. Consequently, a muscle with longer fascicles will involve a greater active volume of muscle and therefore, a greater amount of ATP will be consumed.

### Force-length relationship

It has been known for decades that the isometric force a muscle can produce depends on its average sarcomere length whereby an optimal muscle length exists. Muscle contraction at longer or shorter lengths than this optimal length results in less isometric muscle force (Ramsey and Street, 1940; Gordon et al., 1966). As it relates to E_run_, for a given amount of muscle force required, the necessary level of activation can be minimized if the muscle is operating near optimal length. In keeping the level of activation low, muscle energy cost, and therefore E_run_ can be reduced. At lengths longer and shorter than optimal length, the energy cost for ion transport is relatively higher.

When considering the energetics of muscle contraction, it is also important to include the absolute length of the muscle fascicles. The force of contraction is dictated by the number of cross-bridges engaged in parallel. For each sarcomere in series, the same number of cross-bridges must be engaged. Therefore, the number of sarcomeres in series will affect the energy cost in a proportional way. This cost of increased fascicle length for isometric contractions is countered by the decrease in relative velocity which is achieved with more sarcomeres in series for dynamic contractions.

### Force-velocity relationship

**Figure 2:**
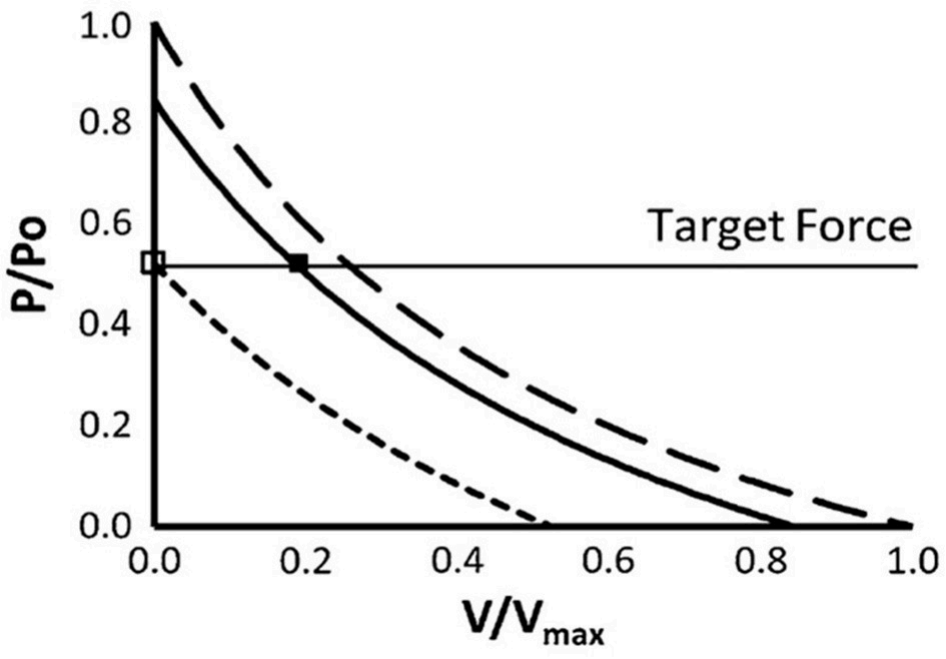
The effect of greater shortening velocity on muscle activation to achieve a target force. The force-velocity relationship, scaled to maximal isometric force (Po) and maximal velocity of shortening (V_max_) (Chow and Darling, 1999). The short dashed and solid lines represent 50 and 85% of maximal motor unit activation, respectively. The long dashed line represents maximal activation. When force can be generated isometrically, target force (P) can be achieved with minimal motor unit activation, as shown by the open square. When shortening is permitted, additional motor unit activation is required (filled square). Used with kind permission of Springer Science+Business Media.

The relationships between mechanical work, efficiency and speed of shortening were first demonstrated by AV Hill almost 100 years ago (Hill, 1922). Since the rate of mechanical work (or power output) is the product of force and velocity, the maximum power output that can be generated by a muscle, or group of muscles, is defined and limited by their force-velocity relationships. The force per cross-bridge apparently decreases linearly with shortening velocity (ter Keurs and de Tombe, 1993), so the hyperbolic shape of the force-velocity relationship is dependent on decreasing numbers of cross-bridges bound as velocity increases. As velocity increases, recruitment must increase to maintain the required force. This has been extensively described previously (Chow and Darling, 1999; MacIntosh and Holash, 2000; Sargeant, 2007), and it is acknowledged that force, not power is the determining factor for muscle activation during running. For a given force requirement, the level of activation and therefore the energy cost, can be minimized if the muscle can operate at a slower shortening velocity (Figure 2).

#### Motor unit recruitment

The muscle's *in vivo* force-length and force-velocity relationships dictate the magnitude of activation required to achieve a given force and velocity of shortening (Praagman et al., 2006) at a given joint configuration (muscle length). The force-velocity relationship dictates that force production for a given level of activation is maximal when that force can be developed isometrically (Fenn and Marsh, 1935; Roberts et al., 1997; Biewener, 1998a) and decreases as shortening velocity increases. Stainsby and Lambert (1979) suggest that the major determinant of metabolic cost of muscle contraction in voluntary movement should be motor unit recruitment. This notion is consistent with the observation that there is a unique bicycling cadence associated with the best efficiency (Coast and Welch, 1985) and this cadence corresponds with the cadence with the lowest magnitude of electromyographic (EMG) activity (MacIntosh et al., 2000). Load, shortening, and velocity of shortening have less impact on the magnitude of energy requirement (Stainsby and Lambert, 1979). For submaximal contractions like those exerted during distance running, the level of activation needed to generate a given force can be minimized when the fascicles are allowed to develop force isometrically.

Keeping the underlying factors dictating energy use in muscle in mind (force, length, velocity, shortening, and activation), we now turn our attention to the energy cost of running. The factors that are not affected by training will be considered first. This will be followed by an examination of those factors which can be affected by training.

## Factors not affected by training

E_run_ can acutely change under the influence of factors other than those related to training. These factors include: environment (wind, temperature, altitude), surface features, footwear, and anthropometry. Each of these factors will be presented in the context of impact on the energy cost of running.

### Environment

#### Wind

The energy required to overcome air-resistance is a function of the runner's frontal surface area and drag coefficient, the air density (altitude, humidity, and pressure) and the relative speed of movement of air past the runner. Pugh (1971) found that work required to overcome air resistance was a linear function of the sum of running speed plus wind velocity, squared. As such, when running at speeds approaching the 2-h marathon barrier (5.8 m·s^−1^) under still wind conditions, the extra energy required to overcome air resistance is ~8% higher compared to running with no air resistance (Pugh, 1970). Presumably the extra energy is required due to the need to generate greater horizontal propelling force. This would relate to the need for increased motor unit recruitment in muscles contributing to the forward propulsion. This extra energy can be nearly abolished by drafting behind other runners, which saves 80% of the extra energy required to overcome wind resistance (Pugh, 1971).

#### Temperature

High environmental temperatures lower a runner's ability to dissipate heat. Increasing heat storage impacts cross-bridge turnover, potentially increasing the energy cost of muscle contraction, and also results in sweating and potentially dehydration. Heat exchange between the body and the environment is achieved by conduction, convection, radiation, and heat loss is also achieved by evaporation (Cheuvront and Haymes, 2001). Any imbalance between heat generated by metabolism and net heat lost by these heat dissipating mechanisms will result in change in heat storage (Bergeron, 2014). Wind serves a thermoregulatory function in that cooler air crosses the skin during running, allowing greater heat loss by convection. This accelerated heat loss will result in lower heat storage for a given speed of running, but heat loss by convection would be considerably less while drafting, so less heat generated through a lower E_run_ will likely be the key feature of a record-attempt at the sub 2-h marathon. A high ratio of surface area to mass will also favor heat loss by conduction, convection, radiation, and evaporation. A lower E_run_ for a given environmental temperature and humidity will result in less heat storage, and a longer exercise duration is permitted. Similarly, a runner with a comparatively low E_run_ can run at a faster speed, for the same rate of heat storage. Where less heat is generated, less energy is consumed by the heart for peripheral circulation of warmed blood from the core to the skin for cooling purposes (Rowell et al., 1969; MacDougall et al., 1974).

### Altitude

Measured at a common absolute speed (255 m·min^−1^), overground sea-level oxygen cost of running is ~4.5% greater than that measured at an altitude of 2,300 m (Daniels et al., 1977). On the treadmill at sea level, there is still 4% greater oxygen cost of running at sea level, so most of the altitude dependent difference is not related to overcoming air resistance. Thus, the energy cost of overcoming air resistance must be only 0.5% of the total energy cost at this speed of running. The only mechanism suggested for the apparent 4% lower oxygen cost at altitude was the possibility of differences in the anaerobic energy contribution at altitude (Daniels et al., 1977). The possibility for anaerobic contribution at altitude when there was not at sea level relates to the compromised maximal oxygen uptake and the lower intensity associated with the anaerobic threshold at altitude. Any contribution by anaerobic metabolism would decrease the oxygen demand, even if the total energy cost was not different.

When converting the oxygen cost to the energy cost, the energy equivalent of the oxygen uptake increases at altitude because of a greater reliance on carbohydrate. For the same energy yield, oxygen uptake would be lower. When running the same absolute speed at altitude as at sea level, this speed may approach the compromised anaerobic threshold, even if it is not exceeded, thus increasing the reliance on carbohydrate.

It was also hypothesized that the thinner air at altitude presents less resistance to ventilation, and therefore a lower work of breathing at altitude. However, Daniels et al. (1977) showed that pulmonary ventilation at altitude was 15–20% greater compared to at sea-level (110 L·min^−1^ vs. 96 L·min^−1^) and it was concluded that there was not a lower energy cost of ventilation. Estimating the energy cost of ventilation at sea-level (96 L·min^−1^) and at altitude (110 L·min^−1^) according to Mazess (1968), yields 0.46 and 0.33 kJ·kg^−1^·km^−1^ at sea-level and altitude, respectively. Thus, the lower work of ventilation at altitude requires 0.13 kJ·kg^−1^·km^−1^ less energy further contributing to the lower E_run_ at altitude when E_run_ is presented as an energy equivalent. Taken together, the differences between sea-level and altitude oxygen cost may likely be explained by the lower work of ventilation and increased energy per liter of oxygen uptake and possible anaerobic contribution, for some of the female subjects, that was not accounted for in the measurement of $\dot{V}O_{2}$ alone.

### Surface features

#### Surface friction

Running straight ahead at a constant speed on a dry, smooth, flat surface requires friction between shoe (or foot) and surface (Frederick, 1986). When on a slippery or wet surface, where the coefficient of friction is reduced, subjects tend to modify their kinematics (and therefore use a less-than-optimal movement pattern) to compensate for soft, energy-dissipating, and uneven surfaces (Frederick, 1983). For example, a reduced coefficient of friction probably involves greater muscle activation prior to footstrike in order to stabilize posture in uncertain circumstances. Presumably, this also elevates E_run_, although further research is required to determine the magnitude of this increase in muscle activation and corresponding increase in energy cost as a result of the less than optimal kinematics caused by lower surface friction.

#### Surface stiffness

E_run_ is also elevated on soft and uneven surfaces such as sand compared to grass or concrete; (Zamparo et al., 1992; Lejeune et al., 1998; Pinnington and Dawson, 2001). The elevated E_run_ on sand has been attributed to a reduction in the re-utilization of elastic energy and/or the energy lost due to backwards translation of the foot during push-off. It has also been hypothesized that an elevated muscle-tendon work while running on sand contributes to the elevated E_run_ (Lejeune et al., 1998). In terms of the muscle energetics presented earlier, these mechanisms (foot slip, increased work and decreased tendon strain energy release) translate to an increased muscle shortening and probably increased motor unit recruitment. It would certainly require increased muscle coactivation for stability when running on an unstable surface. Both of these factors would increase the energy cost of muscle contraction, contributing to increased E_run_.

#### Footwear

The additional mass of footwear predictably increases E_run_ by ~1% per 100 grams of added mass per shoe (Frederick et al., 1984). This 1% increase in E_run_ is fairly consistent across a range of running speeds (Franz et al., 2012) and also degrades running performance (e.g., 3,000 m time-trial time) to a similar extent (Hoogkamer et al., 2016). It has been suggested that a potential mechanism by which footwear might reduce E_run_ is because footwear serves to reduce some of the impact shock. A reduction in E_run_ of 3% with well-cushioned shoes compared to poorly-cushioned ones supports this notion (Frederick, 1986). These authors developed a “cost of cushioning” hypothesis whereby a portion of the measured E_run_ in well-cushioned shoes is reduced because less muscle activation is required to brace for the force of impact with the ground. To support this hypothesis, E_run_ was compared between well-cushioned shod and unshod conditions. The former condition would incur an estimated increase in E_run_ as a result of the mass of the shoes. Despite the added mass of the shoes, E_run_ was not different between shod and unshod conditions.

Stearne et al. (2016) proposed that a portion of the metabolic energy required during running could be saved by the arch spring. These authors demonstrated that restricting foot arch compression increased E_run_ by 6%. This elevated metabolic cost was not seen during walking or incline running, due apparently to the smaller role of elastic energy savings in these gaits. Their results further support the notion that orthotic insoles and arch-support footwear, which are often prescribed to runners, may reduce the foot arch's elastic energy storage and result in an elevated E_run_ while wearing these types of orthotics or shoes (Berg and Sady, 1985).

It has also been speculated that the design of footwear midsole construction may enhance energy return, and therefore E_run_. Worobets et al. (2013) have shown a small (0.9–1.1%) but significant difference in $\dot{V}O_{2}$ between an energy-return midsole (Adidas Boost™) and a conventional ethyl vinyl acetate midsole in runners running below the anaerobic threshold. To eliminate any confounding factors such as shoe construction, Tung et al. (2014) isolated the effect of cushioning on E_run_ by attaching the same cushioning foam to the belt of a treadmill. In so doing, E_run_ was reduced by 1.6% when runners ran unshod on the cushioned belt in comparison to running unshod without the cushioning. Interestingly, E_run_ was not different between shod and unshod conditions on a normal treadmill belt, likely because the beneficial effects of cushioning were balanced by the detrimental effects of added shoe mass. These results suggest (1) shoe mass can have a meaningful influence on the measured E_run_ and (2) there exists a trade-off between running in very light running shoes at the expense of extra cushioning in order to minimize E_run_. Runners are also able to assess shoe comfort reliably (Hennig et al., 1996) and it has been hypothesized that comfort could relate to performance (Nigg, 2001). In fact, oxygen cost was 0.7% lower in shoes deemed “most comfortable” compared to those deemed “least comfortable” (Luo et al., 2009). Further, studies into the specific mechanism for a lower E_run_ with particular reference to muscle energetics associated with some footwear design (kinematics, kinetics, muscle activity etc.) are needed.

### Anthropometry

#### Ankle and foot morphology

E_run_ is determined primarily by the energy needed for muscle contraction of sufficient average force to support body weight for the full stride duration (Kram and Taylor, 1990). Therefore, average muscle force and thus muscle energy cost is related to the average vertical force (F_z_) during stance, as dictated by body mass and the F_z_ moment arm and the moment arm of the Achilles tendon (Ker et al., 1987; Carrier et al., 1994). The ratio of F_z_ moment arm to that of the Achilles tendon is referred to as the gear ratio. Often, the F_z_ moment arm length is interpolated from known forefoot length. In this case, the ratio of forefoot length to AT moment arm is referred to as the foot lever ratio (Kunimasa et al., 2014). The F_z_ moment arm can be altered by changing ankle joint kinematics during the stance phase. This has important implications to E_run_ since changes in joint angle configuration at touch-down result in changes in the F_z_ moment arm. The relative change in the gear ratio for a given F_z_ will determine the magnitude of the required muscle force. Reductions in the gear ratio result in a reduction in muscle forces and this should be reflected as lower energy cost.

**Figure 3:**
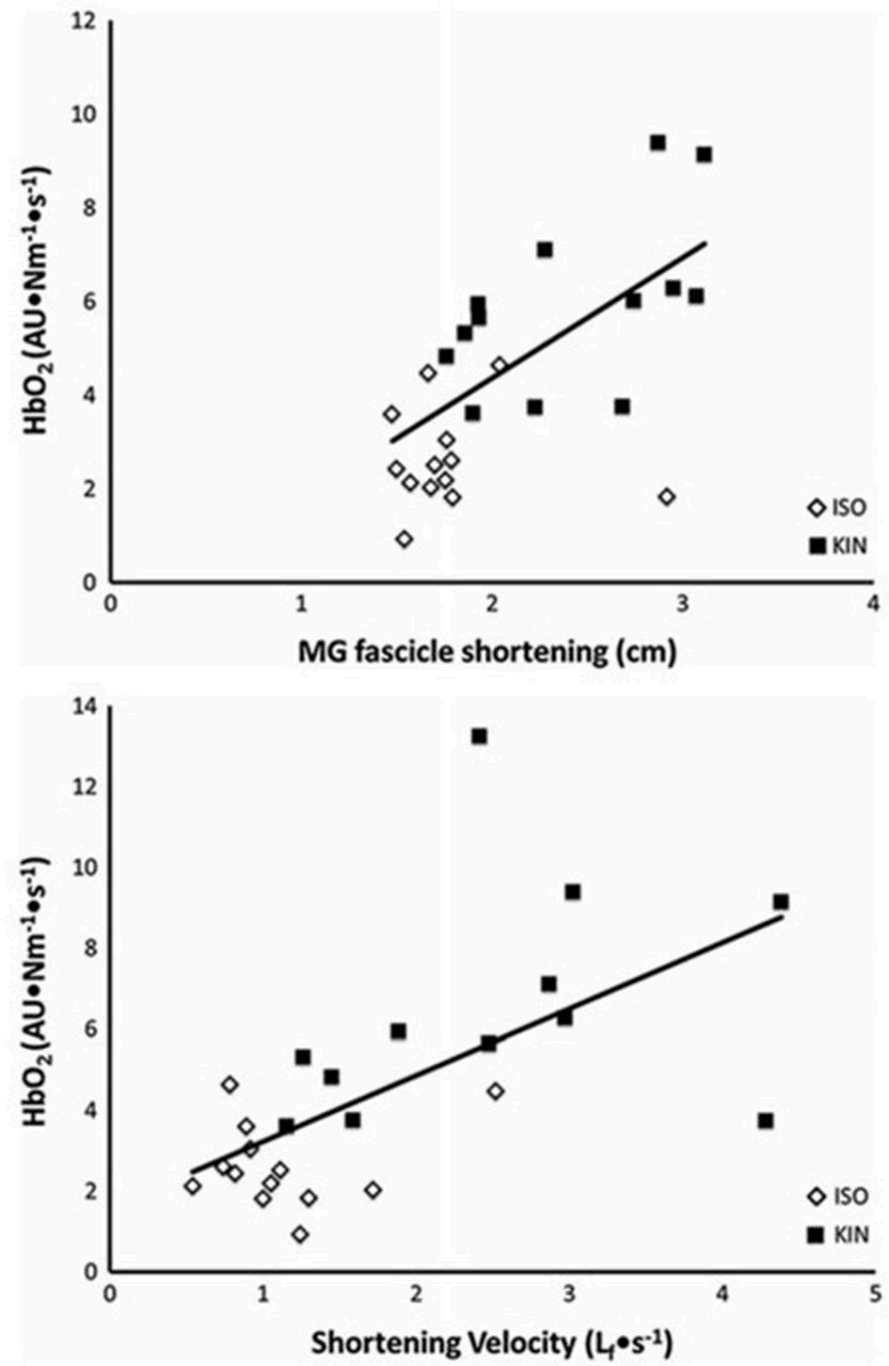
The relationship between the rate of energy use to maintain a given torque (HbO2·impulse^−1^) and magnitude of muscle fascicle shortening (top) and fascicle shortening velocity (bottom). The open diamonds represent measurements made during a fixed-end contraction (ISO). The filled squares represent those measurements made when additional shortening was permitted (KIN). From Fletcher et al. (2013a).

During running, the ankle angle at touchdown is nearly 90°, and the excursion during stance in good runners is nearly 10° more than in elite runners (Cavanagh et al., 1977; Williams and Cavanagh, 1987). A small excursion in the same amount of time translates to lower angular velocity and a corresponding slower velocity of muscle contraction of the ankle plantarflexors. A slower velocity of contraction results in lower level of activation needed to generate a given force and consequently lower energy cost of muscle contraction for these muscles. Using measurements of hemoglobin desaturation during blood flow occluded plantarflexion exercise (which was assumed to be proportional to energy use), Fletcher et al. (2013a) showed that a faster muscle fascicle shortening and/or velocity of fascicle shortening elevates energy use. The relationship between muscle energy cost, shortening, and shortening velocity is shown in Figure 3.

A shorter AT moment arm, measured at rest, has been associated with a lower oxygen cost of running (Scholz et al., 2008; Mooses et al., 2014). The advantage of a short AT moment arm in reducing E_run_ has been attributed to increases in the elastic energy storage/release from the AT during running since larger AT forces for a given joint moment are required with a short AT moment arm; more elastic strain energy is stored and released in a tendon when AT forces are higher. To claim the benefit of a short moment arm is dependent on the estimated extra elastic energy storage ignores the fact that additional muscle energy cost would be required to generate the additional force production of the muscles. This extra muscle energy cost has been estimated to be considerably higher than the extra energy stored in and subsequently released from the tendon (Fletcher and MacIntosh, 2015).

Shorter moment arms also permit slower muscle shortening velocity to achieve a given joint angular velocity (Nagano and Komura, 2003) and require less shortening for a given joint excursion. This slower velocity would permit a higher force without much increase in activation. As previously suggested however, the elevated AT force associated with a shorter moment arm may also incur a substantial muscle energy cost (Fletcher and MacIntosh, 2015) and as such, a longer AT moment arm may help reduce E_run_ by reducing the required muscle force and level of muscle activation to sustain a given joint moment. To support this hypothesis, elite Kenyan long-distance runners, a population known for their exceptionally-low E_run_ (Larsen, 2003; Wilber and Pitsiladis, 2012), have longer AT moment arm lengths and shorter forefoot lengths compared to similarly-trained Japanese distance runners (Kunimasa et al., 2014). van Werkhoven and Piazza (2017) recently found a significant relationship between peak Achilles tendon force and heel length, suggesting subjects with shorter heels experience larger Achilles tendon forces. These larger forces, however, were not associated with a reduced oxygen uptake measured at a common speed for all subjects. These authors, and others contend that while larger Achilles tendon forces should increase tendon stretch and strain energy storage, larger forces also require higher muscle activation and elevated metabolic cost (Perl et al., 2012; van Werkhoven and Piazza, 2017). It remains unclear why these authors did not find the same negative correlation between oxygen uptake and heel length, as has been reported previously (Scholz et al., 2008; Raichlen et al., 2011; Mooses et al., 2014).

The ratio of forefoot to AT moment arm length is known as the foot-lever or gear ratio; a low gear ratio is associated with better E_run_ (Mooses et al., 2014). The gear ratio determines the force and length change needed in the gastrocnemius muscle and these factors will affect the energy cost of muscle contraction. Reducing the gear ratio from 2 to 1.5, by reducing forefoot length and keeping the AT moment arm constant reduces the estimated triceps surae muscle energy cost by nearly 40% (Fletcher and MacIntosh, 2015), assuming the same amount of shortening. Less force is needed if the F_z_ moment arm is shorter. Similarly, reducing the gear ratio by increasing the AT moment arm will also reduce the required muscle force but also necessitates a greater amount of shortening for a given angular displacement so the energy savings would not be this large.

The length of the F_z_ moment arm is dictated by the footstrike pattern and the length of the forefoot. Forefoot length is another anatomical feature (along with presumably short or long moment arm lengths) for which humans have evolved, presumably to favor economical walking and running compared to other primate species. In relation to body mass, humans possess extremely short forefoot lengths (Rolian et al., 2009). This evolutionary adaptation has long been assumed to benefit bipedal locomotion since short toes require smaller plantarflexor forces to balance the large dorsiflexion moments as a result of F_z_ (Weidenreich, 1923; Mann and Hagy, 1979). Using kinematics, force and plantar pressure measurements, this hypothesis was tested in a sample of human subjects (Rolian et al., 2009). It was demonstrated that subjects with relatively long forefoot lengths had to generate more than four times the peak flexor force over a single stance phase compared to short-forefoot individuals. The authors suspected that such an increase in force output would lead to at least a small increase in the metabolic cost of running. This seems very likely given that the elevated muscle force would result in a greater active muscle volume and a concomitant increase in energy cost. Thus, it seems logical to suggest that it is the ratio of Fz moment arm to AT moment arm length, rather than the absolute AT moment arm length itself which dictates the muscle energy cost.

#### Body mass, body composition, and mass distribution

Body composition and distribution of mass may be another relevant feature in dictating muscle energy cost, and therefore E_run_. Active skeletal muscle is primarily responsible for the energy use, so a body mass consisting of a high proportion of skeletal muscle mass and low fat mass should be advantageous in reducing the absolute energy cost of running over a fixed distance (J·m^−1^). This lower absolute energy cost has advantages in less heat generation, and lower relative muscle activation needed for running at a given speed. Although stored fat contributes to the provision of metabolic energy during running, it is considered that in most cases much more than enough energy is available. Transporting metabolically-inactive tissue like fat would come at a metabolic cost. In fact, Kenyan boys have smaller calf circumference than boys of similar age from other continents (Larsen, 2003). This suggests that even lower muscle mass may be advantageous, as long as sufficient muscle mass is available to provide the required forces and support the metabolic rate.

It is estimated that the oxygen cost of running (measured as $\dot{V}O_{2}$, ml·kg^−1^·min^−1^) was elevated by 4.5% for every additional kg of load carried distally on the legs (500 g distributed across both legs) whereas the energy cost was only elevated by 1% when that same mass was carried on the trunk (Jones et al., 1986). Therefore, minimizing the mass of the swinging limbs, by minimizing fat and unnecessary muscle mass in these areas should reduce E_run_, as long as the muscle mass necessary to generate the forces and movements is maintained.

Since running involves rotation of the limbs, a substantial portion of the limb's mass should be located at a close proximity to the joint center of rotation. This serves to minimize the limb moment of inertia. Moving the limbs comprises a substantial portion of the total metabolic cost of running; the joint moment needed to impart an angular acceleration is proportional to the moment of inertia (Fenn, 1930; Cavagna et al., 1964). Swinging the limbs during running should come at a substantial energy cost. Modica and Kram (2005) used a device that assisted the forward swing of the leg, reducing the need of the muscles to swing the leg directly. Their results revealed a reduction in metabolic cost by 20%. This estimate was later refined to ~7% of the metabolic cost of running (Warddrip, 2007), the difference likely a result of the device used by Modica and Kram also aiding in forward propulsion (Arellano and Kram, 2014a).

The metabolic cost of arm swing has also been addressed (Arellano and Kram, 2014b). Swinging the arms incurred a small metabolic cost; however, the arm swing also serves to reduce the amplitude of shoulder and torso rotation. Without arm swing, shoulder and torso rotation must increase to counterbalance the rotational angular momentum created by swinging of the legs. Thus, running with a normal arm swing (i.e., arm carriage with a bent elbow) incurs the lowest E_run_ since moment of inertia of the upper limbs is lowest. The benefit of normal arm swing during running relates to a reduced E_run_ of ~3%. Hypothetically though, E_run_ could be reduced by an even greater extent without the extra mass of the arms, since the reduction in metabolic cost is nearly proportional to the reduced mass (Arellano and Kram, 2014b). Future investigations into the energy cost of elite Paralympic arm amputee distance runners may help resolve this hypothesis.

Above, we have attempted to outline those factors not affected by training which likely alter the energy cost of muscle contraction, and therefore, serve as determinants of whole-body E_run_. There exist specific anthropometric (e.g., limb length) and morphological (e.g., ankle and foot anatomy) characteristics that influence the measured E_run_.

## Factors affecting E_run_ that are altered by training

It is well-known that E_run_ is lower in trained distance runners compared to lesser-trained runners (Pollock, 1977; Morgan et al., 1989; Fletcher et al., 2009) thus it is clear that E_run_ is altered by both short and long-term training protocols. These training strategies have recently been reviewed (Barnes and Kilding, 2015). Below, we outline the various factors affecting E_run_ that are altered by training and consider the influence of these factors on muscle energy cost.

### Anthropometry

#### Body mass

Body mass may or not be a trainable feature. Long-distance runners are shorter and lighter than middle-distance runners (Cavanagh et al., 1977). Also, elite African runners appear to be of lower body mass (Coetzer et al., 1993) and BMI (Saltin et al., 1995) compared to their Caucasian counterparts. These anthropometric differences appear to have persisted since childhood (Larsen et al., 2004). However, body fat mass can also be reduced through large volumes of aerobic and high-intensity training, through increases in caloric expenditure. Also, transporting metabolically-inactive tissue would elevate the energy cost. While little research has examined why low body mass confers an athletic advantage, several factors specifically related to the energy cost of muscle contraction may explain this. For example, it is well-established that F_z_ expressed relative to body mass is increased as a function of running speed (Keller et al., 1996). Thus, at a given running speed, the absolute F_z_ is lower in lighter runners compared to heavier runners. As such, there should be lower energy cost required by the active muscles. Over a wide range of body mass, across different species, Taylor et al. (1980) showed that E_run_ at a particular speed is proportional to the force exerted by the muscles active during stance. By manipulating the required muscle force by the addition of extra weight it was shown that the increased energy cost was proportional to the weight of the carried load.

### Muscle properties

E_run_ at a given speed, is determined by the total active muscle volume and the rate at which that unit volume of muscle transforms energy (Kram and Taylor, 1990; Roberts et al., 1998a). The volume of active muscle is equal to the cross-sectional area (CSA) and the muscle fascicle length of the active motor units. The rate at which each unit volume of muscle uses energy for isometric contractions is related to the muscle fiber type; fast-twitch muscles have higher rates of energy use related to the elevated cost of cross-bridge cycling and activation costs (Rall, 1985; Barclay et al., 2010b). E_run_ increases as a function of running speed since a higher force is developed over a shorter period of time, requiring activation of additional motor units. Faster running speed also requires a faster velocity of shortening. When muscle shortens, additional recruitment is required to maintain force according to the force-velocity relationship. At some critical velocity of muscle shortening, the time-dependent (fiber-type dependent) turnover of cross-bridges becomes inconsequential and the turnover is dictated by the velocity of shortening. This critical velocity will be faster with fast-twitch muscle.

It is well-established that muscle cross-sectional area increases after a period of resistance training which may (Kawakami et al., 1995; Blazevich et al., 2003) or may not (Blazevich et al., 2007; Seynnes et al., 2007) be accompanied by a concomitant decrease in muscle fascicle length, at least in pennate muscle; changes in fascicle length appearing prior to an increase in muscle CSA. However, to run at a given submaximal speed, an increase in absolute strength as a result of increased muscle CSA would result in a lower relative intensity. This lower relative intensity may not require the need to recruit higher threshold motor units, where the muscle energy cost is higher. This may be one of the explanations by which E_run_ is improved following a period of strength training (Balsalobre-Fernandez et al., 2016; Berryman et al., 2017). However, any additional muscle mass that is not used during running results in essentially wasted energy, since carrying that mass will cost energy. Chronic endurance training may also result in a shift to a higher proportion of slow Type I fibers (Rusko, 1992) further contributing to the reduction in muscle energy cost at a given speed.

### Tendon stiffness

Strength training has also been shown to increase tendon stiffness (Kubo et al., 2001a,b; Kubo et al., 2002) and increased Achilles tendon stiffness has been proposed to be one of the main mechanisms behind an improved E_run_ following plyometric training (Saunders et al., 2006) despite the apparent reduction in energy storage and return associated with a stiffer tendon.

It is known that the energy cost of contraction is related to the level of motor unit activation and both the amount of shortening and the shortening velocity (Stainsby and Lambert, 1979). Tendon stiffness can influence the magnitude of shortening and the shortening velocity of the muscle fascicles (Fletcher et al., 2013a), but these parameters can also be affected by the kinematics of the associated joint(s). Several papers reporting on a variety of species and muscle functional tasks highlight the fact that muscle shortening patterns during natural movement are well-matched to take advantage of the muscle's force-length-velocity relationships (Lutz and Rome, 1994; Roberts et al., 1997; Askew and Marsh, 1998). For example, Lutz and Rome (1994) found that the semimembranosus muscle of the frog operated at near-optimal sarcomere lengths and shortening velocities to maximize power output during maximal jumping. This effect would not be possible unless the tendon was perfectly tuned (with respect to stiffness and proportion of muscle-tendon length occupied) to allow the muscle to operate at the appropriate length and velocity. This effect is also true for human running (Kawakami et al., 1998; Ishikawa et al., 2007; Farris and Sawicki, 2012).

The tendon can also act in such a way as to minimize the amount of shortening that is required by the muscle in order to minimize the metabolic cost. At the same level of muscle activation, when muscles shorten, they exert less force than when they contract isometrically but have at least as high a metabolic rate; thus, their economy of force generation is lower (Woledge et al., 1985; Alexander, 1991). By minimizing the length change during active muscle contraction, the tendon allows the muscle's force-length-velocity relationship to be optimized. In theory, if the length change of the whole muscle-tendon unit can be accommodated by the tendon alone, the muscle fibers can operate isometrically, thus minimizing the level of muscle activation required to produce the necessary force as a result of the force-velocity relationship. To test this theory, Holt et al. (2014) determined the cost of force production in frog muscles acting isometrically, and have mechanical energy stored and released by the tendon, compared to muscles undergoing stretch-shortening cycles, as if there was no tendon in-series with the muscle. These authors show that the energy cost of shortening contractions was nearly triple the energy cost of isometric force production. These results were obtained when the energy cost was normalized for force during the contractions. In actual fact, the shortening contraction had a much lower force than the isometric contraction so considering the need to have similar force production during running, additional motor unit recruitment would be needed in the shortening condition as suggested by the results of Fletcher et al. (2013a). This increased motor unit recruitment would contribute to the energy cost difference between isometric and shortening contractions.

The Achilles tendon also accommodates much of the muscle-tendon unit length change during human running (Ishikawa et al., 2007; Lichtwark et al., 2007) thus greatly reducing the shortening-related muscle energy cost (Fletcher and MacIntosh, 2015). Presumably, the mechanical properties of the Achilles tendon are “tuned” to accommodate the majority of muscle-tendon unit length change. Any change in these mechanical properties would affect the magnitude of length change of the muscle fascicles, and energy cost would necessarily be higher if the Achilles tendon cannot accommodate muscle-tendon unit length change.

The relative shortening velocities in the ankle extensors of running turkeys has recently been measured directly in which the above hypothetical scenario has been shown to occur (Gabaldon et al., 2008). During level running, the shortening velocity of the lateral gastrocnemius was quite low (~0.05 V/V_max_), supporting the notion that force can be maximized and activation minimized by low shortening velocities. Having to run up an incline required slightly greater V/V_max_ ratios (~0.12 V/V_max_) and the volume of active muscle that had to be recruited increased in accordance with the muscle's force-velocity properties in order to generate the required force.

If the tendon is too stiff, then lengthening and shortening is required by the fascicles and the volume of active muscle recruitment increases. If the tendon is too compliant, much of the energy for force generation will be consumed shortening the fascicles even with negligible joint rotation. In the case where higher forces need to be generated as running speed increases, too compliant a tendon would require greater fascicle shortening than that necessary for joint rotation, resulting in higher velocity of muscle shortening. This suggests that there may be an “optimal tendon compliance” with respect to minimizing muscle shortening.

### Does an “optimal stiffness” exist to minimize the EC of running?

It has previously been shown that in a group of trained distance runners, the most economical runners displayed a higher Achilles tendon stiffness compared to the less economical runners (Arampatzis et al., 2006; Fletcher et al., 2010). The former study demonstrated the opposite to be true in the patellar tendon—that the most economical runners had a lower patellar tendon stiffness compared to the less economical runners (Arampatzis et al., 2006). This opposite result suggests that the roles of these two muscles in minimizing the energy cost during running are different. We contend that the role of the tendon in running is to minimize the energy cost of muscle contraction. Is it possible that energy cost is minimized in the quadriceps muscles by a more compliant tendon, while a stiffer tendon reduces energy cost in the triceps surae?

There are apparent advantages of stiff tendons in some cases, and compliant tendons in other cases. The lengthening of a tendon for energy storage is relevant in stretch-shortening cycles where a substantial pre-stretch of the tendon occurs early in a contraction. A compliant tendon allows more energy conversion of either kinetic or gravitational energy to potential strain energy. This energy can subsequently be released upon shortening. A compliant tendon may also help by allowing the tendon to lengthen during the stretch phase of the SSC and shorten during the shortening phase, thereby keeping fascicle shortening velocity low and reducing the necessary level of activation of motor units required to generate the force. In situations where power is important, optimal tendon compliance would allow muscle fascicles to shorten at the velocity associated with peak-power output (Askew and Marsh, 1998; Gabaldón et al., 2008) and corresponding shortening of the tendon can contribute to the power generation. This may be the case in the patellar tendon, which would lend support to previous evidence suggesting a more compliant patellar tendon might decrease E_run_ (Albracht and Arampatzis, 2006; Arampatzis et al., 2006).

Conversely, a more compliant AT requires greater muscle fascicle shortening and/or velocity of fascicle shortening for a given joint movement. In the AT, a joint movement may be favored over elastic energy storage and release. This is the case because for a given amount and rate of muscle tendon unit shortening, less muscle fascicle shortening is needed with a stiff tendon compared to a compliant one. The additional fascicle shortening is needed to accommodate tendon stretch as force increases. We have recently estimated the tendon strain energy release from the AT and compared that to the estimated muscle energy cost in order for this strain energy storage to occur (Fletcher and MacIntosh, 2015). These results demonstrate that the storage and release of tendon strain energy comes at a considerable muscle energy cost. Therefore, reducing shortening-induced energy cost contributes to a reduced E_run_.

### Running mechanics

#### Stride length and stride frequency

At speeds below the anaerobic threshold, where E_run_ is most appropriately measured, the lowest E_run_ in humans is generally thought to occur at stride frequencies of 83–91 strides per minute (Hunter and Smith, 2007). The freely-chosen stride frequency closely corresponds to the stride frequency associated with the lowest energy cost (Högberg, 1952; Cavanagh and Williams, 1982; Hunter and Smith, 2007) particularly in trained runners. At a given running speed, a change in stride frequency must result in an opposite change in stride length; however, the naturally chosen stride frequencies and stride lengths, both increase with faster running speeds. There is a proportionally greater increase in stride length compared to the increase in stride frequency, at least at submaximal speeds where the measurement of E_run_ is valid (Cavanagh and Kram, 1989).

Small animals use more energy (per kg of body mass) to run a given distance than do large animals (Kram and Taylor, 1990) since small animals must take many strides to cover the same distance a large animal can cover in one stride. The mass-specific energy cost is highest in small animals since the muscle fascicles of these animals must develop force and relax more quickly, thus requiring greater rates of cross-bridge cycling and Ca^2+^ pumping (Barany, 1967; Rome, 1992). Considering human runners, those runners with longer legs, and thus longer stride lengths should have a lower energy cost; they will take fewer strides to cover a given distance than a runner with small strides. However, the relationship between stride length (expressed either in absolute terms or relative to height or leg length) and E_run_ in human runners, unlike the relationship seen across a wide-range of stride frequencies in animals, is moderate at best (Cavanagh and Williams, 1982; Williams and Cavanagh, 1987; Cavanagh and Kram, 1989).

Running is often considered a bouncing gait whereby humans literally bounce along the ground (Cavagna et al., 1964), storing, and recovering kinetic and potential energy as the center of mass rises and falls with each stride, thus closely resembling a simple spring. To address this, simple studies of humans hopping have revealed optimal conditions for energy storage and release. By having subjects hop at various speeds on a treadmill, Farley et al. (1991) were able to deduce that a range of hopping frequencies existed whereby the body behaved like a spring, storing, and recovering elastic energy. However, at faster than optimal frequencies, the time available to apply force to the ground was necessarily shorter, and more contacts per unit time would be required. At slightly slower than optimal frequency, the body did not behave in a spring-like manner and the recovery of elastic energy was reduced. Clearly there is a trade-off between ground contact time, and the requirement to generate force rapidly and the ability to sustain large forces over a relatively long period during the stance phase, which serves to minimize E_run_.

The fact that runners tend to choose a stride frequency slightly lower than optimal frequency suggests a greater importance is placed on maintaining ground contact time (and thus allowing a lower recruitment of muscle fibers) over maximizing the storage and release of elastic energy. The self-selected stride frequency should be the one at which the metabolic cost of operating the springs is the lowest (Farley et al., 1993) since muscle metabolic energy is required in order to store and release elastic strain energy from the tendons (Alexander, 1986; Fletcher and MacIntosh, 2015). An interesting situation that avoids this issue is that the horse has a vestigial “muscle,” with tendon but no muscle fibers (Biewener, 1998b) that provides a pure energy storage and return without the metabolic cost.

#### Ground contact time

Modeling running as a simple spring-mass system can characterize the mechanics of the body's center of mass quite well (McMahon and Cheng, 1990; Farley et al., 1993); however, it does not adequately explain the energetics of running. Theoretically a perfectly-elastic spring could supply all of the mechanical work on the center of mass during running, and no net metabolic cost would be required (Arellano and Kram, 2014a). A complement to the spring-mass model hypothesis, the “cost of generating force hypothesis” was proposed (Taylor et al., 1980).

By measuring the metabolic cost of carrying various loads, these authors observed the metabolic cost increased in direct proportion to the added load. Therefore, it was proposed that the metabolic cost of running arose in association with the cost of generating force over time, rather than generating mechanical work. The metabolic cost is proportional to the average vertical force applied to the ground and inversely proportional to the ground contact time over which the force can be applied (Kram and Taylor, 1990). The required peak vertical force must be proportionally higher as speed increases, elevating the metabolic cost since muscles must generate more force while shortening at a faster velocity. To generate the higher force, while compensating for the decreased force per motor unit due to the force-velocity relationship, more motor units must be recruited (Roberts et al., 1998b). In further support the cost of generating force hypothesis, several authors have shown an inverse relationship between E_run_ and ground contact time (Williams and Cavanagh, 1987; Chapman et al., 2012; Di Michelle and Merni, 2014). These results suggest the speed-associated increase in E_run_ is a result of the elevated muscle energy cost associated with generating greater peak forces over a shorter period of time.

#### Footstrike pattern

It appears that a rearfoot strike pattern is more economical than either a midfoot or forefoot strike pattern (Gruber et al., 2013; Ogueta-Alday et al., 2014). Heelstrike reduces the plantarflexor moment at the ankle because the center of pressure resides under the heel of the foot during the first half of stance and this reduces the length of the corresponding (F_z_) moment arm (Cavanagh and Lafortune, 1980; Williams and Cavanagh, 1987). Conversely, the center of pressure during stance, a surrogate of the Fz moment arm length, is centered under the ball of the support foot in the forefoot landing pattern. Thus, heel strike pattern is associated with a substantially reduced EMG of the lateral gastrocnemius and soleus muscles compared to forefoot strike (Cunningham et al., 2010). Williams and Cavanagh (1987) found the most economical runners were those with a heelstrike pattern. Forefoot striking may result in a longer F_z_ moment arm, resulting in higher necessary TS force.

The main issue with examining differences in E_run_ between forefoot and heel strike patterns is that many studies artificially impose an unnatural gait on the subject. Thus, a lower E_run_ measured under one condition may be the result of runners being unfamiliar with the novel gait pattern. Gruber et al. (2013) measured E_run_ in habitual forefoot and heelstrike runners and found no difference in $\dot{V}O_{2}$ between groups when running with their habitual footstrike pattern. Interestingly, at all running speeds (3–4 m·s^−1^ was the range evaluated), runners habituated to the heel strike pattern showed a higher $\dot{V}O_{2}$ when asked to forefoot strike, which was not seen when the forefoot group ran with a heel strike pattern. Only at high speeds was the heel strike pattern less economical in the habitual forefoot runners. When the muscle-tendon unit of the triceps surae was modeled to assess the muscle mechanics and energetic differences between foot strike patterns, it was shown that the forefoot strike pattern resulted in a near-isometric contraction during stance. This allows a lower muscle energy cost for a given force compared to the heel strike pattern, where high contraction velocities during stance were demonstrated. A significant difference in the metabolic energy cost, however, could not be shown.

#### Flexibility

Despite the general belief among runners and coaches that greater flexibility may result in improved E_run_ (Craib et al., 1996), there is very little evidence to support this notion. A lower flexibility (measured during a sit and reach test) is associated with a lower E_run_ (Gleim et al., 1990; Craib et al., 1996; Trehearn and Buresh, 2009). Various suggestions have been made by which a lower flexibility may decrease E_run_: (1) reducing the trunk muscle energy cost to maintain stability (Craib et al., 1996) and/or (2) increasing the storage and return of elastic energy (Jones, 2002). The latter mechanism appears unlikely given that such a small amount of mechanical energy is stored and released as elastic energy. This mechanical energy represents only a small savings in total metabolic energy (Ker et al., 1987; Fletcher and MacIntosh, 2015) and mechanically, a stiff AT stores less strain energy for a given force compared to a more compliant tendon. It should be pointed out that if muscle had been required to produce the work done by the tendon, the energy cost would have been 4–5 times greater than the work accomplished by the tendon. As we have previously suggested, an optimal tendon stiffness exists and therefore, a delicate balance between the amount of stretching training (with the intention that stretching training will reduce AT stiffness Kubo et al., 2002; Morse et al., 2008) and strength training [to increase tendon stiffness (Kubo et al., 2001a,b) may result in less than optimal tendon mechanical properties where optimal properties minimize muscle energy cost (Fletcher et al., 2013a)]. Another possible mechanism for low flexibility contributing to low E_run_ is that low flexibility may relate to short muscle fascicles and shorter muscles should use less energy when velocity of shortening is not important.

## Conclusions and future directions: muscle energetics and E_run_

E_run_ has been extensively studied in the biomechanics and exercise physiology literature and is known to be influenced by a variety of factors. However, much of the interpretation of E_run_ exists from the measurement of the steady-state $\dot{V}O_{2}$ at a given submaximal running speed, without calculation of the energy equivalent. Although, this approach has been useful in comparisons between conditions when RER is not much different, it is difficult to conclude with confidence whether previous results showing differences between groups of runners and/conditions (e.g., male vs. female E_run_, altitude vs. sea-level) would still exist had E_run_ been expressed in terms of energy cost to run a fixed distance at a given relative intensity rather than an oxygen equivalent. We recently demonstrated that when E_run_ is measured appropriately, that no sex-related differences in E_run_ exist (Fletcher et al., 2013b; Black et al., 2017). It is only recently that this expression of E_run_ has been encouraged. In any circumstance, when the quantification of E_run_ relies on measurement of $\dot{V}O_{2}$ the intensity must be below anaerobic threshold. Although, we believe it is most appropriate to calculate the energy equivalent of oxygen uptake, there is a considerable body of literature available with valid conclusions where only oxygen cost is presented.

Here, we have reviewed the biomechanical and physiological factors which influence E_run_ from the perspective of muscle energetics. This has allowed us to consider the relative importance of the storage and release of elastic energy from tendon impacting the energy cost, which we argue is relatively minor compared to the muscle energy cost required for muscles in series with the tendons that store the elastic strain energy. Consideration has been given to the influence of biomechanics (limb mass and length, AT, and vertical ground reaction force etc.) and physiology (force-length-velocity properties of muscle) in dictating the muscle energy cost, and therefore determining E_run_.

Future research in elite athletes should be aimed at the effectiveness of different training interventions (e.g., strength, stretching, or plyometric training) on E_run_ expressed in terms of energy. Specifically, a greater understanding of the muscle and tendon interactions during running is warranted; during distance running, where does the muscle operate relative to their submaximal force-length-velocity relationships? How is this altered through training intervention (where muscle and tendon properties may be changed)? What is the impact of fatigue (mechanical or physiological) on the muscle energy cost, and on E_run_ and what is the mechanism of this change?

Future directions should also include the measurement of factors which dictate muscle energy cost across different circumstances that may alter muscle function (aging, disease, disuse) in order to best prescribe appropriate training and/or rehabilitation programs. An interesting special circumstance is consideration of elite Paralympic athletes who may have compromised muscle and/or tendon function or for individuals where exercise tolerance may be limited by an elevated energy expenditure.

## Author contributions

JF and BM were responsible for conception of the review. JF drafted the work. JF and BM revised it critically for important intellectual content. JF and BM approved the final version of the manuscript and both authors agree to be accountable for all aspects of the work.

### Conflict of interest statement

The authors declare that the research was conducted in the absence of any commercial or financial relationships that could be construed as a potential conflict of interest.
